# Maternal Diet Quality, Body Mass Index and Resource Use in the Perinatal Period: An Observational Study

**DOI:** 10.3390/nu12113532

**Published:** 2020-11-17

**Authors:** Zoe Szewczyk, Natasha Weaver, Megan Rollo, Simon Deeming, Elizabeth Holliday, Penny Reeves, Clare Collins

**Affiliations:** 1 Hunter Medical Research Institute (HMRI) Lot 1, Kookaburra Circuit, New Lambton Heights, NSW 2305, Australia; Natasha.weaver@newcastle.edu.au (N.W.); Simon.deeming@hmri.org.au (S.D.); Liz.holliday@newcastle.edu.au (E.H.); Penny.reeves@hmri.org.au (P.R.); 2 School of Medicine and Public Health, The University of Newcastle, University Drive, Callaghan, NSW 2308, Australia; 3 School of Health Sciences, The University of Newcastle, University Drive, Callaghan, NSW 2308, Australia; Megan.rollo@newcastle.edu.au; 4 Priority Research Centre for Physical Activity and Nutrition, The University of Newcastle, University Drive, Callaghan, NSW 2308, Australia

**Keywords:** dietary assessment, pregnancy, nutrition, economic evaluation, directed acyclic graphs (DAGs), maternal and infant

## Abstract

The impact of pre-pregnancy obesity and maternal diet quality on the use of healthcare resources during the perinatal period is underexplored. We assessed the effects of body mass index (BMI) and diet quality on the use of healthcare resources, to identify whether maternal diet quality may be effectively targeted to reduce antenatal heath care resource use, independent of women’s BMI. Cross-sectional data and inpatient medical records were gathered from pregnant women attending publicly funded antenatal outpatient clinics in Newcastle, Australia. Dietary intake was self-reported, using the Australian Eating Survey (AES) food frequency questionnaire, and diet quality was quantified from the AES subscale, the Australian Recommended Food Score (ARFS). Mean pre-pregnancy BMI was 28.8 kg/m^2^ (range: 14.7 kg/m^2^–64 kg/m^2^). Mean ARFS was 28.8 (SD = 13.1). Higher BMI was associated with increased odds of caesarean delivery; women in obese class II (35.0–39.9 kg/m^2^) had significantly higher odds of caesarean delivery compared to women of normal weight, (OR = 2.13, 95% CI 1.03 to 4.39; *p* = 0.04). Using Australian Refined Diagnosis Related Group categories for birth admission, the average cost of the birth admission was $1348 more for women in the obese class II, and $1952 more for women in the obese class III, compared to women in a normal BMI weight class. Higher ARFS was associated with a small statistically significant reduction in maternal length of stay (RR = 1.24, 95% CI 1.00, 1.54; *p* = 0.05). There was no evidence of an association between ARFS and mode of delivery or “midwifery-in-the-home-visits”.

## 1. Introduction

Obesity in pregnancy has become a major challenge for obstetric care in high-income countries [1]. Approximately 50% of women who become pregnant have overweight (body mass index (BMI) > 25 kg/m^2^–30 kg /m^2^) or obesity (BMI > 30 kg/m^2^) [1], and the prevalence of obesity is rising [2]. High pre-pregnancy BMI has been strongly associated with excessive gestational weight gain [3], incidence of gestational diabetes mellitus, pre-eclampsia, pre-term delivery [3], large-for-gestational-age infants, caesarean delivery [4], miscarriage, antepartum stillbirth, complications at delivery and increased postpartum weight retention [1,3,5]. Given the elevated risk to the mother and infant, obstetric and midwifery clinical practice guidelines recommend that healthcare facilities have well-defined pathways for the care of women with obesity, with increased care and monitoring relative to the antenatal care pathways of non-obese women [6,7]. This has resource use implications for the healthcare system. Clinical practice guidelines also provide “healthy eating in pregnancy” recommendations to address knowledge related to risk of diet-related conditions such as obesity [6]. However, there are no routine implementation interventions ensuring that clinical practice guideline recommendations for healthy eating in pregnancy are translated into practice [6]. This is a concern, as many Australian women fail to meet nationally recommended nutrient targets and do not appear to improve their diet quality when planning to become pregnant, or during pregnancy [8,9]. The economic implications of poor maternal nutrition, and its relationship with BMI and the use of healthcare resources (henceforth referred to as healthcare-resource use) is underexplored [10]. 

A recent World Health Organisation report, titled *Promoting Health and Preventing Disease: An Economic Case*, identified that improved maternal nutrition was as a potentially cost-effective target for health-promotion strategies aiming to improve maternal and infant health outcomes [11]. The volume of services and total expenditure on the delivery of maternity services means that relatively minor improvements in the cost per maternity patient could generate significant cost savings to public hospitals [12]. In particular, antenatal nutrition and gestational weight gain were identified as targets for health-promotion interventions aiming to improve maternal weight status and reduce demand on the healthcare system [13]. A recent study of infants born to mothers with overweight or obesity in the United Kingdom found that the usage rate for all healthcare services was significantly greater in infants born to mothers with obesity than infants born to mothers with healthy weight [14]. Infants born to mothers with obesity experienced a 39% higher rate of inpatient admissions and a 55% longer duration of inpatient stays, utilising, on average, 72% more resource costs [14]. Similarly, a cross-sectional comparative study of the short- and long-term effects of gestational diabetes mellitus (GDM) on healthcare costs found GDM was independently associated with an average additional cost of €817.60 (€2012) during pregnancy, due to additional delivery and neonatal care costs and an additional €680.50 in annual infant healthcare costs two to five years post-pregnancy [15]. A modelled economic evaluation exploring the short-term costs of maternal overweight, gestational diabetes and related macrosomia was conducted by Lenoir-Wijnkoop et al. [16] and found the average total additional costs for overweight was estimated to be $18,290 (USD) per pregnancy/delivery, which consists of an additional $13,047 for mothers with overweight and $5243 for their infants. Maternal diabetes was associated with an additional $15,593 per pregnancy/delivery, while foetal macrosomia was a significant risk factor for the development of obesity in childhood [16]. While overweight and obesity in women of child-bearing age and their offspring are of international concern, less attention has been paid to the economic consequences. At present, the cost of nutrition related perinatal health outcomes is unknown [10]. The range of potential targets for antenatal health promotion interventions, including nutrition interventions, is extensive, and healthcare-decision makers face growing pressure to optimize value, as well as quality, of healthcare [17]. 

Ensuring evidence-based healthcare is effective, as well as efficient and equitable, is critical if governments are to succeed in realising improved population health outcomes and contained per capita healthcare expenditure [18]. To identify technologies, interventions and models of care that provide the greatest value, healthcare providers are increasingly using health economic analyses to inform evidence-based decision-making [19]. Applied health economic evaluation informs evidence-based decision making by assisting healthcare-decision makers “identify, measure, and value activities with the necessary impact, scalability, and sustainability to optimize population health” [20]. High-quality cost and effectiveness data are a prerequisite for evidence-based decision-making. The highest cost of routine maternity care is incurred during the admission for birth (76%), followed by the non-admitted healthcare provided during the antenatal (17%) and postnatal (6%) periods [12]. However, the breakdown of these costs by population group is unknown. There is also insufficient evidence of the cost of nutrition interventions in pregnancy [10]. Given this absence of evidence, data on maternal dietary intake, obesity and their relationship with healthcare-resource use is needed to inform research, guidelines and decision makers of the economic impacts of current antenatal health promotion and clinical practice [21]. To address these evidence gaps, a cross-sectional population-based study was designed to quantify specific perinatal-healthcare-resource use associated with maternal weight status and diet quality in a sample of pregnant women attending a public hospital in New South Wales, Australia. The hypothesis was that high BMI and low diet quality would be associated with increased healthcare-resource use, with diet quality potentially having a direct effect, independent of BMI. The aims of this study were as follows:

- Assess the diet quality of pregnant Australian women attending a public hospital antenatal clinic;
- Estimate the total effect of BMI, adjusted for diet quality, on healthcare-resource use during the delivery admission, including mode of delivery, length of stay, admission to intensive care and midwifery-in-the-home service;
- Estimate the total effect of maternal diet quality on healthcare-resource use during the delivery admission;
- Estimate the direct effect of maternal diet quality on healthcare-resource use during the delivery admission.

## 2. Materials and Methods 

### 2.1. The Study

The study was an observational cross-sectional study in which patients attended public hospital antenatal outpatient clinics for routine antenatal care and were managed according to current clinical practice. The target sample size was 600 women with complete diet-quality scores, which were informed by investigator experience and feasibility. The study was advertised in the local newspaper and disseminated across university social media. Posters and fliers advertising the study were placed in the antenatal clinic, satellite clinics and birthing packs. Patients were also invited to complete the survey whilst in the waiting room, prior to their antenatal appointment, by trained volunteers. 

All subjects gave their informed consent for inclusion before they participated in the study. The study was approved by the University of Newcastle Human Research Ethics Committee, Australia, study reference number H-2017-0101. Hunter Area Research Ethics Committee in August 2016 reference number HREC/16/HNE/189. The reporting adhered to the Strengthening the Reporting of Observational Studies in Epidemiology (STROBE) guidelines.

#### 2.1.1. Study Population and Setting 

Pregnant women aged 18 years or older, at 28–36 weeks of gestation (third trimester), and planning to deliver at the John Hunter Hospital were eligible to participate in the study. The time period of 28–36 weeks of gestation was selected, since the tool selected to measure diet, the Australia Eating Survey (AES), assesses intake over the previous three to six month, and we had previously shown significant correlations between dietary intake in early and late pregnancy [9]. The John Hunter Hospital, located in the Hunter New England Local Health District, New South Wales, Australia, is a large (550 bed) tertiary referral hospital, delivering around 4000 babies each year [22]. Participants were not excluded based on illnesses or known medical conditions.

#### 2.1.2. The Survey 

Self-reported demographic, health and diet quality data were collected at baseline (recruitment), and medical records data for the delivery admission were collected after discharge of mother and infant. The baseline survey consisted of four components: (1) consent and participant information statement; (2) participant information; (3) demographic data; and (4) the AES and could be completed in about 25–35 min. All subjects gave their informed consent for inclusion before they participated in the study. Study data were collected and managed, using REDCap electronic data capture tools hosted at The University of Newcastle [23,24].

#### 2.1.3. Study Recruitment

Trained volunteer research personnel (University students enrolled in the final years of a Bachelor of Nutrition and Dietetics) recruited study participants from the clinic between March 2018 and November 2018. All personnel undertook a mandatory workshop and further in-clinic training alongside a project officer. A brief and informative script was used by research personnel, to verbally screen women for eligibility, inform women of the survey content and purpose, and invite women to participate. Consenting participants then completed the survey on a tablet via the REDCap offline mobile application. Women at less than 28 weeks of gestation were invited via email to complete the survey when they reached 28 weeks’ gestation. Women unable to complete the survey in the clinic due to fatigue, distractions (e.g., other children or feeling unwell) or being called to their appointment were emailed the remainder of their survey for later completion. An automated reminder email was sent seven days later, to all participants who had not finished the survey. All study participants had given birth by January 2019.

### 2.2. Statistical and Economic Analyses

The economic analysis took a healthcare provider’s perspective to identify, measure and value outcomes associated with the provision of routine healthcare in the delivery period. The analysis excluded costs to patients and society. Since the time horizon for inclusion of relevant healthcare-resource use is set at less than 12 months, conversion or discounting of costs was not required [25].

#### 2.2.1. Identification and Measurement of Exposure and Outcomes

Diet quality was quantified, using the previously validated Australian Recommended Food Score (ARFS) [26,27,28], derived from a subset of questions from the AES food frequency questionnaire for adults [26]. The AES is a 120-item semi-quantitative food-frequency questionnaire that was designed to assess usual dietary intake of individuals aged 18 years or older, based on a list of foods most commonly eaten by Australians. The AES has undergone comprehensive evaluation for validity and reliability, reported elsewhere [26]. The total ARFS score is calculated by summing the points for foods that are aligned with the core foods in the Australian Guide to Healthy Eating consumed at least weekly, with a total score ranging from 0 to 73 [26,27,28]. A higher score reflects greater alignment with recommendation in the Australian Dietary Guidelines.

Maternal clinical outcomes and healthcare-resource use from the delivery admission and associated home healthcare, Maternity Home Services, was collected from hospital databases using individual patient medical record numbers (MRN). Specific healthcare-resource use required for the management of maternal obesity was identified from the literature and reviewed by content experts (see Appendix A: Table A1). For the purpose of the current analyses, healthcare-resource use is defined as follows:

- Mode of delivery: caesarean versus vaginal (natural, instrumental, breech, compound).
- Maternal length of stay: (count in days).
- Maternal admission to intensive care: (yes or no).
- Midwifery-in-the-home service utilisation: total number of follow-up care visits associated with maternal discharge post-delivery (count).

Establishing associations between an intervention target and an outcome is a mandatory precursor to economic evaluation [19]. For the current study, if associations between BMI or diet quality and mode of delivery or admission to intensive care were established, healthcare-resource use was then defined and costed, using the Australian Refined Diagnosis Related Group (AR-DRG) classification system for admitted acute episodes of care in Australian public and private hospitals. The AR-DRG codes classify units of hospital output and group inpatient stays into clinically meaningful categories at similar levels of complexity (outputs) and consuming similar resources (inputs) [29]. Independent Hospital Pricing Authority national weighted activity unit (NWAU) calculators are used to estimate cost of care based on AR-DRG classifications. All costs were reported in 2020, Australian dollars ($AUD).

Mode of delivery and admission to intensive care have specific AR-DRG classifications. However, length of stay and midwifery-in-the-home care visits are non-clinical variables that do not have a diagnostic criterion. As such, length of stay and midwifery-in-the-home were reported in clinically relevant natural units, days and total number of visits, respectively.

#### 2.2.2. Development and Use of Causal Diagrams

Many nutrition research studies aim to identify and quantify causal relationships between nutrition and health outcomes [30]. The limitations of traditional methods for assessing associations in observational studies and inferring causality are widely recognised [31]. However, the use of experimental design in the antenatal period needs careful ethical and practical consideration [31]. In order to investigate causality, observational data must be interrogated carefully, with attention to the potential for known and unknown confounders and other biases [31]. Incorrect casual inferences are more likely to occur in observational studies than clinical trials, due to confounding bias [31]. A common way to control for confounding bias in an observational study is to include confounders as covariates in a regression model; however, careful consideration of which variables should be adjusted for is required [30]. Adjustment is needed to ensure that the effect estimate for the exposure of interest is unconfounded. It is commonly believed that it is necessary to control for all potential confounders and that adjusting for more confounders cannot worsen causal inference; however, the inclusion of unnecessary covariates, or over-adjustment, carries the risk of introducing unintended bias and reducing statistical power [32].

For the current study, there exist complex preconception processes influence maternal and infant health outcomes and healthcare-resource use, and these may also influence diet quality (see Appendix A: Table A1). To depict the presumed causal relationships between the exposure, outcome and potential confounding variables related to the exposure and/or outcome, directed acyclic graphs (DAGs) were developed, using existing evidence (listed in Appendix A: Table A1) and expert opinion. DAGitty, a browser-based environment for creating, editing and analysing causal diagrams (DAGs) [33], was used to create a DAG, to visually depict the direct or total effects of interest for each aim: Aims (ii), (iii) and (iv). The three DAGs are included in the Supplementary Materials, along with the DAGitty code to reproduce them and the potential minimum adjustment sets that were identified.

A facility of DAGitty is its analysis of the DAG and provision of candidate “minimum adjustment sets” for estimating unconfounded effects of interest. Each adjustment set is minimal in the sense that it is sufficient to remove confounding bias for the effect of interest and includes no unnecessary variables. The inclusion of unnecessary covariates can reduce efficiency or introduce unintended bias. For a given effect of interest, there are potentially multiple minimum adjustment sets, any one of which could be used. The identified adjustment sets for each aim are listed below:

- Aim (ii) adjustment set: maternal age, maternal education, parity and ARFS.
- Aim (iii) adjustment set: maternal education,
- Aim (iv) adjustment set: maternal age, maternal education and BMI.

#### 2.2.3. Statistical Methods

For Aim (i), the diet quality of pregnant Australian women attending a public hospital antenatal outpatient clinic was measured by using a diet-quality index and reported as total ARFS score, using descriptive statistics (mean with standard deviation or median with range for continuous variables, and frequency with percent for categorical variables). For estimating the effects in Aims (ii), (iii) and (iv), regression models were fitted within a generalized linear modelling (GLM) framework, with response distribution and link function as appropriate for each response.

Caesarean delivery was modelled by using logistic regression, assuming a binomial response distribution (for caesarean versus vaginal birth), and using the logit link function. Logistic models were estimated with Firth’s penalised Maximum Likelihood [34], to reduce bias in parameter estimates due to data sparsity involving some response/explanatory variable combinations. Maternal length of stay and number of midwifery in the home visits were modelled as count responses, using Poisson regression with the log link function. Overdispersion was assessed by using hypothesis tests for the dispersion parameter. The proportion of participants admitted to higher-level care (0.6%) was too rare to perform regression analyses for this outcome.

During the modelling process, fit statistics were examined, to assess whether categorical variables could be simplified by combining categories. Based on the Akaike Information Criterion (AIC) and Likelihood Ratio Test (LRT), maternal education was reduced from seven categories to a binary variable (university versus not), and parity was reduced from a count variable to a binary variable indicating primiparous (parity = 0) versus not (parity > 0). We also considered reducing the number of BMI categories; however, based on an increased AIC and significant LRT, the six-level variable was retained. ARFS was rescaled (into quintiles) to aid interpretation of effect estimates. The validity of using ARFS quintiles has been reported elsewhere [28].

Results are reported as exponentiated parameter estimates with 95% Wald confidence intervals accompanied by *p*-values from Wald tests. Statistical significance was declared at the conventional 0.05 level, to two decimal places for all analyses. Data manipulation and statistical analyses were performed by using SAS 9.4 (SAS Institute, Cary, NC, USA)™ software.

## 3. Results

### 3.1. Study Recruitment

**Figure 1:**
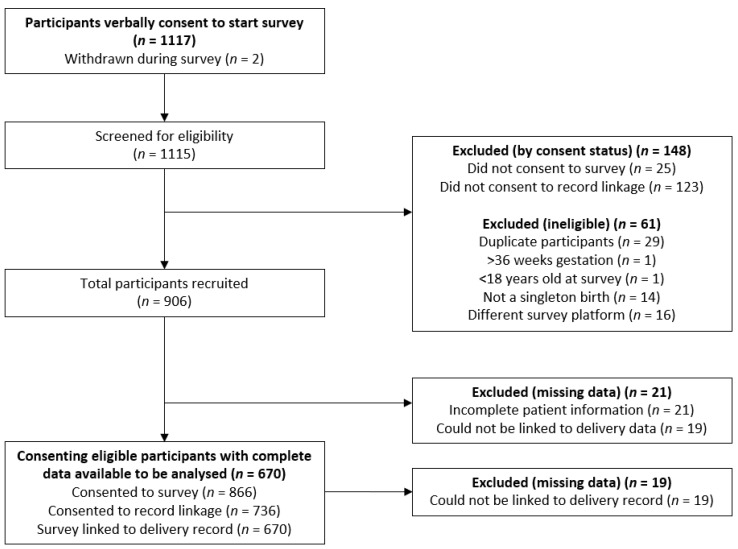
Strengthening the Reporting of Observational Studies in Epidemiology (STROBE) flow diagram of participant inclusion and exclusion.

A total of 1117 individuals commenced the survey (see Figure 1). Of these, two withdrew during survey completion. A total of 148 did not consent to participate or partially completed the consent questions, and 61 participants did not meet the eligibility criteria. A total of 670 consenting participants were eligible to participate and were linked to medical records data.

### 3.2. Participant Demographics

The mean age of participants was 30 years (range: 18.4–53.0), and the mean gestation length at time of survey was 32 weeks. Most participants were born in Australia (90%) and spoke English at home (93%). A total of 6.8% of participants identified as Aboriginal or Torres Strait Islander. A total of 85% of participants were married or in a de facto relationship, 11% were single mothers and 3.3% were divorced or separated. The most frequent level of educational attainment was ≤year 12 (or equivalent) level of education (37.1%). The most frequent annual household income category was ≥$104,000 (27%), and a further 25% of participants reported incomes of $65,000 to $104,000. The mean pre-pregnancy BMI was 28.8 kg/m^2^ (range: 14.7 kg/m^2^–64 kg/m^2^), with 59% of participants having overweight or obesity, 37% having normal weight and 4.5% having underweight. Just over half (54%) of participants said they had received pregnancy diet advice from a health professional during the current pregnancy. Table 1 summarises study participant demographic and health data.

### 3.3. Aim (i): Diet Quality of Pregnant Women

Diet quality was assessed using the ARFS, with a mean ARFS of 28.8 (SD 13.1) points. The mean ARFS for those with a pre-pregnancy BMI in the normal weight category was 31.2 (SD 13.1). The mean ARFS was lower for women outside the normal BMI category, and ranged from 27.1 to 28.3 points (Table 2). 

### 3.4. Aim (ii): Estimate of the Total Effect of BMI on Healthcare-Resource Use

Results from the analyses investigating the total effect of BMI on specific healthcare-resource use are shown in Table 4. The mean gestational age at birth was 38.4 weeks (SD 1.4 weeks), and 93% of infants were delivered at term (>37 weeks). The most common birth type was normal vaginal birth (50%), a further 40% of the babies were delivered via caesarean section and 10% had an abnormal vaginal birth (including instrumental, breech and compound birth). Four women required higher level care or were admitted to intensive care (refer to Table 3).

The mean maternal postnatal length of stay was 2.1 (SD 1.6) days, and the median was 2.0 (range: 0.0, 9.0), inclusive of the four women admitted to intensive care. The mean length of stay for women in the normal BMI weight class was 1.9 (SD 1.6) days and was slightly lower in the underweight BMI category, at 1.6 (SD 1.5) days. Amongst overweight and obese women, the mean length of stay was 2.1 (SD 1.6) in the overweight class and 2.2 (SD 1.6) for women in the BMI category obese class III. The mean number of midwifery-in-the-home care visits was 1.6 (SD 0.9), and the median was 2.0 (range: 0.0, 6.0). The mean number of midwifery-in-the-home care visits for women in the overweight category was 1.6 (SD 0.9), and the range did not vary substantially across pre-pregnancy BMI categories obese class I–III (1.55–1.5).

Women in the overweight and obese categories had increased odds of caesarean delivery, relative to women in the normal BMI category. The magnitude of this effect increased with increasing BMI category. The association for obese class II (35.0–39.9 kg/m^2^) reached 0.05 significance (OR = 2.13, 95% CI 1.03 to 4.39; *p* = 0.04), indicating that women in obese class II had about double the odds of caesarean delivery, compared to women with normal BMI (Table 4).

The association was very similar for women in obese class III, who also had about a two-fold higher odds of caesarean delivery (OR 1.92; 95% CI 0.98 to 3.73; *p* = 0.056).

A total of 666 patients had AR-DRG classification for their birth admission. Of these, there were 242 (99%) women in the normal weight category and 62 (98%) women in obese class II and III.

The AR-DRG cost for birth admission did not vary by length of stay or maternal age. In general, there was a higher rate of complex deliveries among women with obesity. Among women in the normal BMI category, 32% had a caesarean delivery and 11% had a birth classified as having “major complexity”. Rates were higher for women in obese class II, with 50% having caesarean delivery and 18% having a “major complexity” birth. Rates were slightly higher again for women in obese class III, with 53% having caesarean delivery and 25% having a “major complexity” birth.

We have reported and compared costs of delivery for women with normal BMI (reference) and women in obese class II and obese class III, due to high similarity of effect estimates and likely clinical importance of results for both obesity classes (see Table 5). The average cost per patient for women in normal weight was $7962. The average cost per patient for women in obese class II was $9309. The incremental difference in admitted patient cost was $1348. That is, in this sample, the birth admission for women in BMI category Obese class II cost $1348 more than women in normal weight class. The average cost of the delivery admission for women in obese class III was $9914, which was $1952 more than for women in the normal weight class and $605 more than women in obese class II.

### 3.5. Aim (iii): Estimate of the Total Effect of Maternal Diet Quality on Healthcare-Resource Use

Results from the analyses investigating the total effect of maternal diet quality on healthcare-resource use during the delivery admission are shown in Table 4. There were no significant effects of ARFS on mode of delivery or the number of midwifery-in-the-home visits a patient required. Women in ARFS Quintile 1 had a 20% increase in the mean length of stay relative to Quintile 5 (RR 1.20; 95% CI 1.00 to 1.44; *p* = 0.05). That is, women with poor diet quality had an increase in average length of stay, when compared to women with the highest level of diet quality.

### 3.6. Aim (iv): Estimate of the Direct Effect of Maternal Diet Quality on Healthcare-Resource Use

Results from the regression analyses investigating the direct effect of maternal diet quality on healthcare-resource use during the delivery admission are shown in Table 4. Women in ARFS Quintile 1 had a 27% increase in the mean length of stay relative to Quintile 5 (RR 1.27; 95% CI 1.05 to 1.53; *p* = 0.01). That is, independent of a woman’s BMI, those with an ARFS score in Quintile 1 (lowest diet quality) had a 27% increase in average length of stay when compared to women with an ARFS score in Quintile 5 (highest diet quality). There was no significant direct effect of ARFS on caesarean delivery or midwifery in the home visits.

Given there was no statistically significant association between ARFS and mode of delivery, admission to intensive care and midwifery in the home visits, analysis of the economic impact of ARFS on these outcomes was not conducted.

## 4. Discussion

This observational study sought to quantify specific perinatal-healthcare-resource use associated with maternal weight status and diet quality, in a sample of pregnant women attending a public hospital in NSW, Australia. It was hypothesized that high BMI and low diet quality would be associated with increased healthcare-resource use, with diet quality potentially having a direct effect, independent of BMI. This study found the odds of caesarean delivery was about two-fold higher for women in obese class II than for women of normal weight. In this sample, the effect size for the association between BMI category obese class III was very similar, but did not quite reach the nominal 0.05 significance threshold (OR 1.92; 95% CI 0.98 to 3.73; *p* = 0.056). With consideration for the real-world impacts of BMI on healthcare-resource use, given similarity of the effect sizes, in a larger sample size, both obese class II and III would likely have achieved statistical significance. Based on these findings and evidence-based guideline recommendations for increased routine monitoring for women classified as obese [6], the impact of both obese class II and obese class III on caesarean delivery is expected to be clinically important. As such, the impact of both obese class II and III on average inpatient cost was explored.

AR-DRG classifications include an estimate of case complexity, which is a classification system within the AR-DRG classifications, to “better explain the variation in costs occurring in the admitted patient data within the AR­DRG classification” [35]. There were higher rates of caesarean delivery and cases with “major complexities” for women in BMI category obese class II and III, relative to women with a BMI in the normal-weight category. The birth admission for women in BMI category obese class II cost $1348 more than women in normal-weight class. The average cost of the birth admission for women in obese class III was $1952 more than for women in the normal-weight class and $605 more than for women in obese class II. Within the perinatal period alone, small improvements in maternal pre-pregnancy BMI could deliver substantive economic benefits to the healthcare system and community. Aside from the economic impact, obesity and increased case complexity have procedural complications for clinicians and the healthcare system. For example, complications from anaesthesia are higher in obese patients compared to normal weight patients [36]. There is increased risk of incorrectly placing an epidural in obese patients as the distance to the epidural space is greater with increased BMI [36,37], risk of difficult intubation is increased in obese patients, monitoring and positioning obese patients under anaesthesia can also pose specific challenges [36]. Obesity is also associated with an increased risk of maternal mortality and anaesthesia-related maternal mortality [37]. From a midwife’s perspective, a 2011 study of midwives and other health professionals caring for obese childbearing women in NSW, Australia, found midwives were concerned about the rapid impact of the obesity epidemic on maternity services and that study participants felt increased pressure in the management of obese pregnant women and the complications associated with their BMI [38]. Pre-pregnancy public health interventions to reduce maternal pre-pregnancy BMI may prevent the onset or mitigate complications in the delivery period and reduce the obesity related risks to mothers, clinicians and the healthcare system.

This study also found that diet quality had a direct effect on maternal length of stay, independent of BMI. Women in ARFS Quintile 1 had a 27% increase in the mean length of stay relative to Quintile 5 (RR 1.27; 95% CI 1.05 to 1.53; *p* = 0.01). That is, independent of a woman’s BMI, those with an ARFS score in Quintile 1 (lowest diet quality) had a 27% increase in average length of stay when compared to women with an ARFS score in Quintile 5 (highest diet quality). The method in which diet quality acts on length of stay is also unknown. The investment required to improve maternal diet quality is unknown [10]. Further investigation is required, given that poor dietary patterns are common among this population [39] and that current systematic review indicate interventions to improve maternal BMI and pregnancy outcome show inconsistent finding in regard to cost-effectiveness [40,41]. This study found no significant association between pre-pregnancy BMI and maternal length of stay or midwifery-in-the-home care visits. Analyses also showed that maternal diet quality had no direct effect on caesarean delivery or midwifery-in-the-home care visits. Greater understanding of the economic impact of maternal-health behaviours and specific dietary components on healthcare-resource use and health outcomes is warranted [10].

### Strengths and Limitations

The limitations of traditional methods for assessing associations in observational studies and inferring causality are widely recognised [31]. In order to investigate causality, observational data must be interrogated carefully, with attention to the potential for known and unknown confounders and other biases [31]. Use of DAGs in observational nutrition research allows for stronger causal inferences, as compared to conventional statistical adjustments alone. A strength of the current study was the extensive DAG development process informed by the relevant literature, and expert opinion to inform assumptions underpinning the statistical and economic models.

This observational study was conducted in the John Hunter Hospital, NSW, where admitted inpatient-cost data are not stored in administrative hospital datasets and, hence, were outside the data available for this analysis. In the absence of individual patient-cost data, the AR-DRG classification was used as a proxy for admitted-inpatient costs [29]. For the purpose of future research, individual patient-cost data may provide greater specificity regarding the association between patient outcomes, resource use and cost. The John Hunter Hospital antenatal outpatient clinic services high-risk patients requiring ongoing management of GDM, pre-eclampsia, those who have had previous adverse outcomes, women with babies in breech position or those who are attending the clinic drug and alcohol services or Indigenous health services. A limitation of the current study is that the sample of women is expected to have worse health outcomes and thus higher healthcare-resource use compared to the broader population of pregnant Australian women. The current analysis also did not allow for data linkage across all service providers. The John Hunter Hospital has five satellite antenatal clinics that patients can attend, but radiology and pathology can be performed at the hospital, in public or private clinics, and patients may attend private general practitioners, specialists and care providers throughout the antenatal period. Data for health service provision prior to delivery were also unavailable. The inherent recall bias associated with retrospective self-report surveys is recognised as a limitation. Further, the AES food frequency questionnaire, although previously used in pregnancy [42], has not been validated in this population group, and, therefore, the findings of this study need to be interpreted in this context.

## 5. Conclusions

The current study aimed to quantify specific perinatal-healthcare-resource use associated with maternal weight status and diet quality, in a sample of pregnant women attending a public hospital in New South Wales, Australia. This study found that the odds of caesarean delivery more than doubled for those in obese class II relative to normal weight women, with pre-pregnancy BMI positively associated with an increased risk of caesarean delivery. On average, the birth admission for women in BMI category obese class II costs $1348 more than women in normal weight class, and women in obese class III cost $1952 more than women in the normal weight class. Both obese classes II and III had a higher incidence of caesarean section and complex cases, compared to women in the normal weight class. Our analyses showed that, independent of a woman’s BMI, those with an ARFS score in Quintile 1 (lowest diet quality) had a 27% increase in average length of stay when compared to women with an ARFS score in Quintile 5 (highest diet quality). Maternal-diet quality had no direct effect on caesarean delivery or midwifery-in-the-home care visits. Poor dietary patterns are common during pregnancy [39]; thus, interventions to improve maternal BMI and diet quality could deliver substantive economic benefits to the healthcare system and community.

## Figures and Tables

**Table 1:** Summary of study participant demographic and health data.

Participant Demographic and Health Data		
Characteristic	Statistic or Class	Total (*N* = 670)
Age at survey	mean (SD)	30.3 (5.5)
	median (min, max)	30.1 (18.4, 53.0)
Aboriginal or Torres Strait Islander	No	600 (93%)
	Yes	44 (6.8%)
Born in Australia	No	62 (9.6%)
	Yes	581 (90%)
Marital status	Married/de facto	548 (85%)
	Divorced/separated	21 (3.3%)
	Single	73 (11%)
Language spoken at home	English only	598 (93%)
	Other	44 (6.9%)
Highest educational qualification	No formal qualifications	20 (3.1%)
	Year 10 or equivalent	107 (17%)
	Year 12 or equivalent	111 (17%)
	Trade/Apprenticeship	29 (4.5%)
	Certificate/Diploma	176 (27%)
	University undergraduate	151 (23%)
	University postgraduate	50 (7.8%)
Annual household income	Less than $20,800	32 (5.1%)
	$20,800 to less than $41,600	44 (7.0%)
	$41,600 to less than $65,000	68 (11%)
	$65,000 to less than $104,000	158 (25%)
	$104,000 or more	172 (27%)
	Not provided	153 (24%)
Weeks of gestation at survey	mean (SD)	32 (3)
	median (min, max)	31 (28, 36)
Received pregnancy diet advice from health professional	Yes	325 (54%)
	No	263 (44%)
	Unsure	15 (2.5%)
Pre-pregnancy body mass index (BMI) measured	mean (SD)	28.8 (8.3)
	median (min, max)	26.8 (14.7, 64.0)
	Underweight (<18.5 kg/m^2^)	30 (4.5%)
	Normal (18.5–24.9 kg/m^2^)	247 (37%)
	Overweight (25.0–29.9 kg/m^2^)	139 (21%)
	Obese Class I (30.0–34.9 kg/m^2^)	116 (17%)
	Obese Class II (35.0–39.9 kg/m^2^)	64 (9.6%)
	Obese class III (≥40 kg/m^2^)	74 (11%)
Number ANC visits	mean (SD)	12.1 (5.3)
	median (min, max)	11.0 (1.0, 40.0)
Alcohol risk score	mean (SD)	0.1 (0.5)
	median (min, max)	0.0 (0.0, 9.0)
Number term pregnancies	mean (SD)	1.3 (1.1)
	median (min, max)	1.0 (0.0, 8.0)
Number preterm pregnancies	mean (SD)	0.1 (0.4)
	median (min, max)	0.0 (0.0, 3.0)
Number living children	mean (SD)	1.3 (1.1)
	median (min, max)	1.0 (0.0, 10.0)
History of endocrine disease	No	534 (80%)
	Yes	136 (20%)
History of hypertension	No	606 (90%)
	Yes	64 (9.6%)
Maternal risk factor—diabetes	No	488 (73%)
	Yes	182 (27%)
Maternal risk factor—hypertension	No	607 (91%)
	Yes	63 (9.4%)
Maternal risk factor—anaemia	No	448 (67%)
	Yes	222 (33%)
Maternal risk factor—smoke during pregnancy	No	568 (85%)
	Yes	102 (15%)

**Table 2:** Maternal-diet quality, measured using the Australian Recommended Food Score (ARFS), and specific healthcare-resource use by BMI category (*N* = 670).

Characteristic	Statistic or Class	Underweight (*n* = 30)	Normal (*n* = 247)	Overweight (*n* = 139)	Obese Class I (*n* = 116)	Obese Class II (*n* = 64)	Obese Class III (*n* = 74)
Diet quality (ARFS)	mean (SD)	27.2 (13.8)	31.2 (13.1)	27.2 (14.3)	27.1 (12.7)	28.3 (9.8)	28.2 (12.9)
	median (min, max)	28.0 (4.0, 56.0)	34.0 (1.0, 54.0)	30.0 (1.0, 50.0)	29.0 (2.0, 52.0)	29.5 (9.0, 46.0)	29.0 (1.0, 51.0)
Maternal length of stay (days)	mean (SD)	1.6 (1.5)	1.9 (1.6)	2.1 (1.6)	2.2 (1.5)	2.2 (1.6)	2.2 (1.6)
	median (min, max)	1.0 (0.0, 5.0)	2.0 (0.0, 9.0)	2.0 (0.0, 7.0)	2.0 (0.0, 8.0)	2.0 (0.0, 9.0)	2.0 (0.0, 7.0)
Number of “midwifery-in- the-home” visits	mean (SD)	1.8 (1.0)	1.6 (0.9)	1.6 (0.9)	1.6 (0.9)	1.6 (0.7)	1.5 (0.8)
	median (min, max)	2.0 (0.0, 5.0)	2.0 (0.0, 6.0)	2.0 (0.0, 4.0)	2.0 (0.0, 4.0)	2.0 (0.0, 3.0)	2.0 (0.0, 3.0)
Delivery mode	Vaginal birth	21 (70%)	167 (68%)	79 (57%)	65 (56%)	33 (52%)	36 (49%)
	Caesarean section	9 (30%)	80 (32%)	60 (43%)	51 (44%)	31 (48%)	38 (51%)

**Table 3:** Participant demographics and healthcare-resource use summary statistics.

Characteristic	Statistic or Class	Total (*N* = 670)
Infant birthweight (grams)	mean (SD)	3359.4 (515.1)
	median (min, max)	3390.0 (1450.0, 4830.0)
Gestational age at birth (weeks)	mean (SD)	38.4 (1.4)
	median (min, max)	38.0 (31.0, 41.0)
Maternal length of stay (days)	mean (SD)	2.1 (1.6)
	median (min, max)	2.0 (0.0, 9.0)
Mode of delivery	Normal vaginal birth	334 (50%)
	Caesarean section	269 (40%)
	Abnormal vaginal birth	67 (10%)
Pre-term birth (<37 weeks)	No	626 (93%)
	Yes	44 (6.6%)
Gender of infant	Male	326 (49%)
	Female	344 (51%)
Birthweight category	Low birth weight (<2500 g)	35 (5.2%)
	Normal range	568 (85%)
	Macrosomia (>4000 g)	67 (10%)
Midwifery-in-the-home care visits	mean (SD)	1.6 (0.9)
	median (min, max)	2.0 (0.0, 6.0)
Maternal admission to higher level care (intensive care)	No	664 (99%)
	Yes	4 (0.6%)

**Table 4:** Estimates of the effect of diet quality and pre-pregnancy BMI on healthcare-resource use.

	Caesarean Delivery		Maternal Length of Stay		MITH Visits	
	Odds Ratio (95% CI)	*p*-Value	Rate Ratio (95% CI)	*p*-Value	Rate Ratio (95% CI)	*p*-Value
Aim (ii)—total effect of BMI *						
Underweight	0.58 (0.16 to 2.08)	0.40	0.78 (0.49 to 1.23)	0.28	0.95 (0.63 to 1.44)	0.82
Normal	(ref)		(ref)		(ref)	
Overweight	1.57 (0.91 to 2.71)	0.11	1.04 (0.86 to 1.26)	0.71	0.95 (0.78 to 1.18)	0.66
Obese Class I	1.18 (0.65 to 2.16)	0.58	1.07 (0.87 to 1.32)	0.51	0.89 (0.70 to 1.12)	0.31
Obese Class II	2.13 (1.03 to 4.39)	0.04	1.11 (0.87 to 1.42)	0.41	0.99 (0.75 to 1.30)	0.92
Obese class III	1.92 (0.98 to 3.73)	0.06	1.10 (0.87 to 1.39)	0.41	0.90 (0.69 to 1.16)	0.41
Aim (iii)—total effect of ARFS **						
Quintile 1	1.08 (0.64 to 1.85)	0.77	1.20 (1.00 to 1.44)	0.05	1.02 (0.83 to 1.26)	0.85
Quintile 2	1.16 (0.69 to 1.96)	0.58	1.10 (0.91 to 1.32)	0.33	1.09 (0.89 to 1.34)	0.41
Quintile 3	0.72 (0.42 to 1.24)	0.24	1.05 (0.87 to 1.27)	0.60	1.01 (0.82 to 1.25)	0.91
Quintile 4	0.93 (0.54 to 1.62)	0.80	1.12 (0.92 to 1.35)	0.26	0.99 (0.79 to 1.22)	0.90
Quintile 5	(ref)		(ref)		(ref)	
Aim (iv)—direct effect of ARFS ***						
Quintile 1	1.23 (0.71 to 2.16)	0.46	1.27 (1.05 to 1.53)	0.01	1.00 (0.81 to 1.24)	0.99
Quintile 2	1.25 (0.72 to 2.17)	0.43	1.14 (0.94 to 1.37)	0.19	1.07 (0.87 to 1.32)	0.52
Quintile 3	0.74 (0.42 to 1.29)	0.29	1.07 (0.88 to 1.29)	0.50	1.00 (0.81 to 1.24)	0.96
Quintile 4	0.97 (0.55 to 1.71)	0.92	1.13 (0.93 to 1.37)	0.21	0.98 (0.78 to 1.21)	0.83
Quintile 1	(ref)	.	(ref)	.	(ref)	.

* Adjusted for ARFS, maternal age, maternal university education (yes versus no) and primiparous (yes versus no). ** Adjusted for maternal university education (yes versus no). *** Adjusted for BMI, maternal age and maternal university education (yes versus no). (ref): reference category used.

**Table 5:** Maternal birth admission Australian Refined Diagnosis Related Group (AR-DRG) classification (with description and price ($AUD, 2020) and mean cost per patient for study participants in BMI categories normal and obese class II and III.

AR-DRG			Normal		Obese Class II		Obese Class III	
Code	Description	NWAU Cost	*n* * = 242	Cost ($) **	*n* = 62	Cost ($) **	*n* = 72	Cost ($) **
O01A	Caesarean delivery, major complexity	$17,170	5	$85,850	2	$34,340	10	$171,700
O01B	Caesarean delivery, intermediate complexity	$12,310	39	$480,090	14	$172,340	15	$184,650
O01C	Caesarean delivery, minor complexity	$10,074	34	$342,516	15	$151,110	13	$130,962
O02A	Vaginal delivery with operating room procedures, major complexity	$12,691	3	$38,073	0	$0	0	$0
O02B	Vaginal delivery with operating room procedures, minor complexity	$9119	6	$54,714	3	$27,357	0	$0
O60A	Vaginal delivery, major complexity	$8967	19	$170,373	9	$80,703	8	$71,736
O60B	Vaginal delivery, intermediate complexity	$6206	82	$508,892	15	$93,090	22	$136,532
O60C	Vaginal delivery, minor complexity	$4560	54	$246,240	4	$18,240	4	$18,240
Cost per patient ***				**$7962**		**$9309**		**$9914**

* Number of participants with AR-DRG available. ** Cost ($) = number of participants × NWAU cost (by AR-DRG classification). *** Cost ($) per total number of patients, by BMI category. NWAU: National Weighted Activity Unit.

## Supplementary Materials

The following are available online at https://www.mdpi.com/2072-6643/12/11/3532/s1, Identifiable healthcare data were used in this study and not appropriate for circulation.

## Appendix A

**Table A1:** List of characteristics collected via patient medical records and evidence of association on preconception process influencing health outcomes and healthcare-resource use.

Study Data	Description	Preconception Process
**Demographics**		
Maternal age	Age in years	- Increased incidence of maternal hypertension and gestational diabetes mellitus, non-elective caesarean delivery and instrumental delivery and preterm delivery and neonatal intensive care admission among mothers of advanced maternal age [43,44,45]. - Mothers of advanced maternal age have increased pregnancy risk when compared to younger mothers [43,46].
Education	Maternal education level acquired: high school, TAFE, tertiary education, post-graduate	- Lower-educated women are more likely to smoke, have passive smoking exposure, have low health control beliefs, and not attend antenatal classes or take supplements [47]. - Low educational attainment has been associated with higher rates of pre-pregnancy obesity [48].
Partner status	Relationship status: single, married, de facto, divorced	- Partner (marital) status has been used previously in antenatal diet quality studies [8,49], serving as a participant specific metric for socioeconomic advantage and disadvantage [50].
Insurance status	Health insurance status: no insurance, private health insurance, private insurance without obstetrics	- The inter-sector difference in obstetric practice [51,52] and maternal and infant health outcomes are well documented [53,54]. - Studies have shown increased rates of caesarean section and pre-labour caesarean section amongst patients with private health insurance [51,55].
**Lifestyle**		
Cigarette smoking	Did the mother smoke nicotine during this pregnancy?	- Antenatal smoking is strongly correlated with preterm birth, low birth weight and adverse infant outcomes [56].
Alcohol consumption	AUDIT-C score	- Antenatal alcohol and substance use are strongly correlated with adverse maternal and infant health outcomes [57,58]. - Older women were significantly more likely than younger women to report drinking while pregnant, but equally likely to reduce their consumption when they became pregnant as their younger counterparts [59].
Diet Quality	Maternal ARFS during current pregnancy	- Suboptimal eating patterns during pregnancy contribute to EGWG, gestational hypertension, pre-eclampsia, GDM, pre-term birth, low and high birth weight, birth defects and still birth [8,9].
**Previous Medical History**		
Body mass index	At booking visit (20 weeks gestation)	- Clinical practice guidelines recommend that healthcare facilities have well-defined pathways for the care of pregnant obese women, with increased monitoring and management in comparison to the pathways for the care of healthy-weight women [7]. - High pre-pregnancy BMI has been shown to be strongly associated with EGWG [3] and increased resource use in the antenatal period [3,60,61]. - There is a linear trend between maternal pre-pregnancy BMI and risk for both elective and unplanned caesarean section [62,63].
Previous mode of delivery	Total number of previous caesarean sections	- Attempting vaginal birth after a previous caesarean section, or repeat elective caesarean section, carries additional risks to the mother and baby [64].
Parity	Number of previous pregnancies	- Parity has been associated with advanced maternal age, sociodemographic status and educational attainment [45].
Assisted reproductive therapy required? (ART)	Did the mother require ART or IVF to conceive this pregnancy?	- Perinatal risks that may be associated with assisted reproductive technology (ART) and ovulation induction include multifetal gestations, prematurity, low birth weight, small for gestational age, perinatal mortality, caesarean delivery, placenta previa, abruptio placentae, preeclampsia and birth defects [65].
Diabetes	Has the mother been diagnosed with type I or type II diabetes?	- Patients with pre-gestational diabetes (types 1 and 2) are more prone to higher rates of pre-eclampsia, prematurity and caesarean section. - Pregnancy may accelerate maternal and infant complications of diabetes [66].
**Recent Antenatal Period**		
Weight change	Did the patient gain an appropriate amount of weight during pregnancy?	- EGWG are risk factors for GDM, pregnancy-induced hypertension and pre-eclampsia, venous thrombo-embolism, labour induction and caesarean delivery [67].
Hypertensive disorders	Was the mother diagnosed with hypertensive disorders?	- High maternal-diet quality may reduce the risk of gestational hypertension for the mother [49]. - Current clinical guidelines for management of hypertensive disorders in pregnancy recommend diagnosis of hypertensive disorders “should lead to increased observation and vigilance” [68].
Gestational diabetes:	Was the mother diagnosed with GDM?	- Prevalence is affected by maternal factors such as history of previous GDM, ethnicity, advanced maternal age, family history of diabetes, pre-pregnancy weight and EGWG [66]. - Potential maternal complications during pregnancy and delivery include pre-eclampsia and higher rates of caesarean delivery, birth injury and postpartum haemorrhage [66]. - For the neonate, complications can include macrosomia (large for gestational age) growth restriction, birth injuries, respiratory distress, hypoglycaemia and jaundice [66]. - GDM is diagnosed at any time throughout the pregnancy, and management includes a prescriptive diet which is expected to be different from the mother’s diet pre-GDM diagnosis [69].
Plurality	Number of infants born (2, 3, 4, …, x)	- Guidelines advise of additional care that should be offered to women with twin and triplet pregnancies above that are routinely offered to all women during pregnancy [70].
Gestation	Number of weeks at delivery	- Gestational age at birth is an important predictor or infant mortality and length of stay [71].
Infant birth weight	Infant birth weight in grams	- Birthweight is a key indicator of infant health and a principal determinant of infant mortality [50]. - Factors that contribute to low birthweight include extremes of maternal age, illness during pregnancy, low socioeconomic position, multiple pregnancy, maternal history of spontaneous abortion, harmful behaviours such as smoking or excessive alcohol consumption, poor nutrition during pregnancy and poor antenatal care [50]. - Low birth weight is a risk factor for inadequate foetal development and amplified risk of chronic disease throughout life [50].
Mode of delivery	Caesarean; surgical intervention (including internal manoeuvres); vaginal birth	- It is considered self-evident that the cost of caesarean delivery is more expensive than natural birth. - Even amongst similar cases, the charges associated with mode of delivery vary widely [72]
Mother length of stay	Mothers length of stay in days	- It is considered self-evident that length of stay and admission to intensive care accrues higher healthcare-resource use and total cost of admission than those without. - Neonatal service levels range from no planned service, Level 1 to Level 6 [73]. Level 6 neonatal care is provided in specialist children’s hospitals where neonatal surgery and complex genetic and metabolic services are located. It is considered self-evident that Level 6 care will accrue higher costs than Level 1 care, due to the complexity of care provided.
Infant length of stay	Infant length of stay in days	
Infant admission to nursery	Neonatal intensive care admission	

ART, Assistive reproductive therapy; AUDIT-C, Alcohol Use Disorders Identification Test; EGWG, excessive gestational weight gain; GDM, gestational diabetes mellitus; TAFE, Technical and Further Education.
