# Supplementary material for: Maternal Diet Quality, Body Mass Index and Resource Use in the Perinatal Period: An Observational Study
Source: Nutrients. 2020 Nov 17;12(11):3532. doi: 10.3390/nu12113532 (PMC7698580; doi:10.3390/nu12113532)
Supplement: Supplementary file 1 [file nutrients-12-03532-s001.pdf]

Supplementary content to be viewed along with the methods section of the manuscript:

There are three roles that variables can assume in a DAG: confounder, mediator, or collider. A confounder is ancestor to both the exposure and the outcome, where an ancestor is a variable that has temporal precedence and a suspected causal influence. For example, in Figure 2, diet quality is a confounder with respect to the relationship between the exposure BMI and the outcome resource use. An unconfounded estimate of the direct effect of BMI on resource use can thus be calculated by adjusting statistically for diet quality in this simplified situation. On the other hand, with respect to the relationship between diet quality as the exposure and the outcome resource use, BMI is a mediator which is a variable that is on a causal pathway leading from the exposure to the outcome. The third type of variable is a collider, which is a variable that is causally influenced by two other variables and lies on a path starting with an arrow leading to the exposure and ending with an arrow leading to the outcome. Crucially, colliders should not be adjusted for statistically as doing so can bias the estimate of the effect of interest [32].

Figure 1. is the colour-coded legend provided by DAGitty to inform model building.

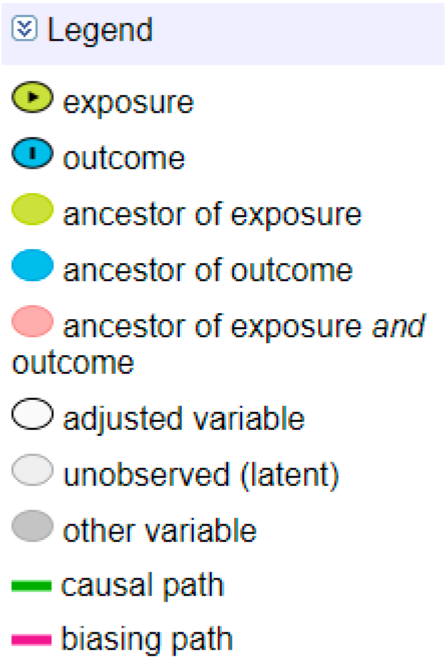

Figure 1. Legend provided in DAGitty used to inform development of DAGs.

Finally, the use of DAGs in causal inference relies on the assumption that the DAG is correct and that no variables with a causal influence have been omitted. There is a further assumption that adjusting for a given confounder completely reduces its bias, which may not be true if residual bias exists due to imperfect measurement of the confounder [32].

Supplementary File

Aim ii) estimate the total unconfounded effect of BMI on specific health care resource use during the delivery admission

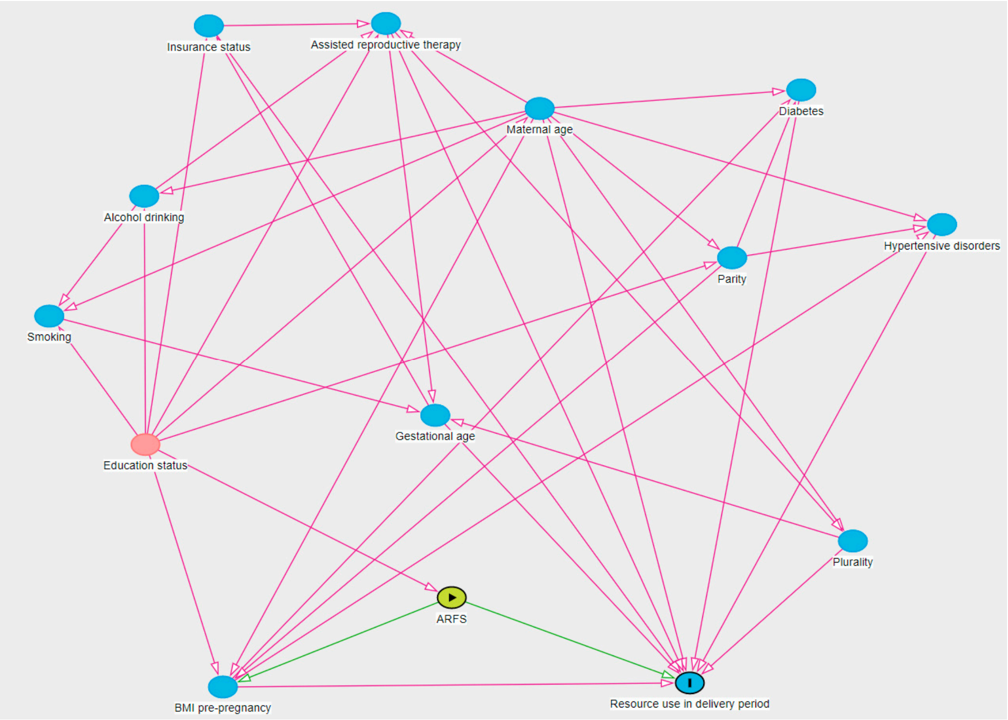

Figure 2. Directed acyclic graph developed for Aim (ii).
Each variable included in the DAG is listed in alphabetical order: 'Alcohol drinking indicates the participants alcohol consumption during this pregnancy; 'ARFS' indicates the participants diet quality; 'Assisted reproductive therapy' (ART) indicates the use for the current pregnancy; 'BMI pre-pregnancy' indicates the mothers BMI pre-pregnancy; 'Diabetes' indicates the participant's history of diabetes and diabetes status for the current pregnancy; 'Education status' indicates the mother's highest level of education attainment; 'Gestational age' indicates to the infants gestational age at birth; 'Hypertensive disorders' indicates the participant's history of hypertensive disorders and status for the current pregnancy; 'Insurance status' indicates a participant's insurance status for the birth admission (public or private patient); 'Maternal age' indicates the mothers age at delivery; 'Parity' indicates the number of pregnancies the participant has had; 'Resource use in the delivery period' indicates the participants resource use for the delivery admission; 'Smoking' indicates the participants smoking status during this pregnancy. Evidence to support the inclusion of each variable in this DAG is include in manuscript Appendix A. Table 5.

To generate the DAG in Figure 3. Launch DAGitty in your browser (http://www.dagitty.net/) and use the following model code:

```
dag {
bb="0,0,1,1"
"Alcohol drinking" [pos="0.179,0.309"]
"Assisted reproductive therapy" [pos="0.385,0.096"]
"BMI pre-pregnancy" [exposure,pos="0.280,0.912"]
```

```
"Education status" [pos="0.168,0.647"]
"Gestational age" [pos="0.427,0.580"]
"Hypertensive disorders" [pos="0.859,0.344"]
"Insurance status" [pos="0.234,0.099"]
"Maternal age" [pos="0.516,0.201"]
"Resource use in delivery period" [outcome,pos="0.644,0.910"]
ARFS [pos="0.452,0.782"]
Diabetes [pos="0.735,0.205"]
Parity [pos="0.680,0.385"]
Plurality [pos="0.783,0.735"]
Smoking [pos="0.098,0.457"]
"Alcohol drinking" -> "Assisted reproductive therapy"
"Alcohol drinking" -> Smoking
"Assisted reproductive therapy" -> "Gestational age"
"Assisted reproductive therapy" -> "Resource use in delivery period"
"Assisted reproductive therapy" -> Plurality
"BMI pre-pregnancy" -> "Hypertensive disorders"
"BMI pre-pregnancy" -> "Resource use in delivery period"
"BMI pre-pregnancy" -> Diabetes
"Education status" -> "Alcohol drinking"
"Education status" -> "Assisted reproductive therapy"
"Education status" -> "BMI pre-pregnancy"
"Education status" -> "Insurance status"
"Education status" -> "Maternal age"
"Education status" -> ARFS
"Education status" -> Parity
"Education status" -> Smoking
"Gestational age" -> "Insurance status"
"Gestational age" -> "Resource use in delivery period"
"Hypertensive disorders" -> "Resource use in delivery period"
"Insurance status" -> "Assisted reproductive therapy"
"Insurance status" -> "Resource use in delivery period"
"Maternal age" -> "Alcohol drinking"
"Maternal age" -> "Assisted reproductive therapy"
"Maternal age" -> "BMI pre-pregnancy"
"Maternal age" -> "Hypertensive disorders"
"Maternal age" -> "Resource use in delivery period"
"Maternal age" -> Diabetes
"Maternal age" -> Parity
"Maternal age" -> Plurality
"Maternal age" -> Smoking
ARFS -> "BMI pre-pregnancy"
ARFS -> "Resource use in delivery period"
```

Diabetes -> "Resource use in delivery period"
Parity -> "BMI pre-pregnancy"
Parity -> "Hypertensive disorders"
Parity -> Diabetes
Plurality -> "Gestational age"
Plurality -> "Resource use in delivery period"
Smoking -> "Gestational age"
}

Minimal sufficient adjustment sets for estimating the total effect of BMI pre-pregnancy on Resource use in delivery period:

- *ARFS, Assisted reproductive therapy, Diabetes, Gestational age, Hypertensive disorders, Insurance status, Maternal age, Plurality*
- *ARFS, Assisted reproductive therapy, Diabetes, Gestational age, Hypertensive disorders, Insurance status, Maternal age, Smoking*
- *ARFS, Diabetes, Education status, Hypertensive disorders, Maternal age*

Aim iii) Estimate the total unconfounded effect of maternal diet quality on resource use during the delivery admission

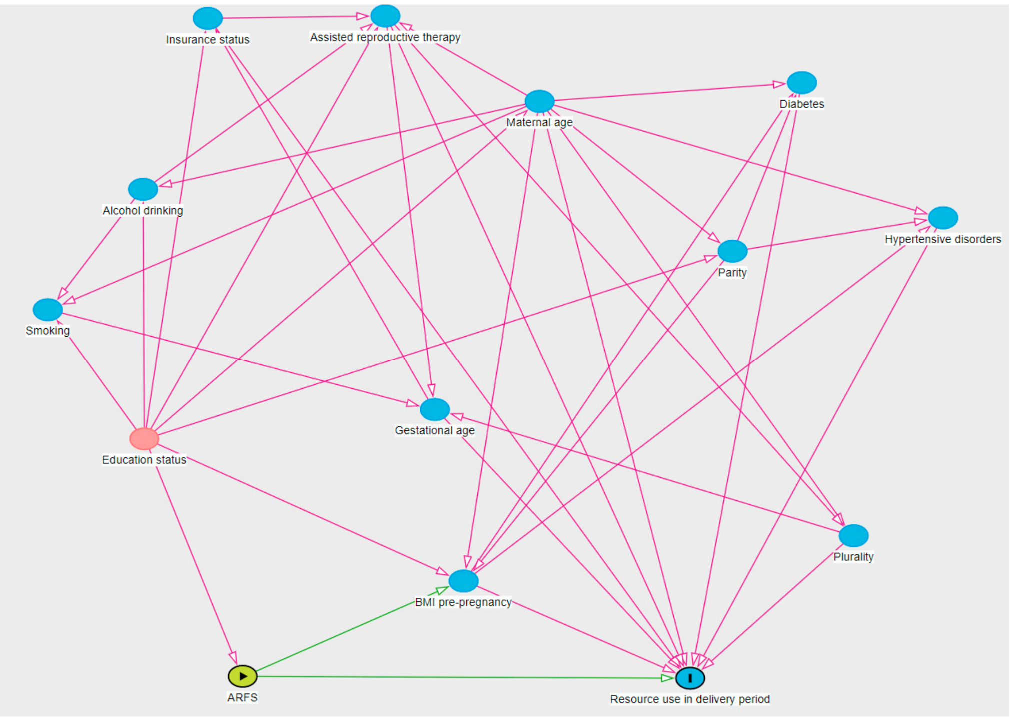

Figure 3. Directed acyclic graph developed for Aim (iii).
Each variable included in the DAG is listed in alphabetical order: 'Alcohol drinking indicates the participants alcohol consumption during this pregnancy; 'ARFS' indicates the participants diet quality; 'Assisted reproductive therapy' (ART) indicates the use for the current pregnancy; 'BMI pre-pregnancy' indicates the mothers BMI pre-pregnancy; 'Diabetes' indicates the participant's history of diabetes and diabetes status for the current pregnancy; 'Education status' indicates the mother's highest level of education attainment; 'Gestational age' indicates to the infants gestational age at birth; 'Hypertensive disorders' indicates the participant's history of hypertensive disorders and status for the current pregnancy; 'Insurance status' indicates a participant's insurance status for the birth admission (public or private patient); 'Maternal age' indicates the mothers age at delivery; 'Parity' indicates the number of pregnancies the participant has had; 'Resource use in the delivery period' indicates the participants resource use for the delivery admission; 'Smoking' indicates the participants smoking status during this pregnancy. Evidence to support the inclusion of each variable in this DAG is include in manuscript Appendix A. Table 5.

To generate the DAG in Figure 3. Launch DAGitty in your browser (http://www.dagitty.net/) and use the following model code:

```
dag {
bb="0,0,1,1"
"Alcohol drinking" [pos="0.179,0.309"]
"Assisted reproductive therapy" [pos="0.385,0.096"]
```

```
"BMI pre-pregnancy" [pos="0.451,0.791"]
"Education status" [pos="0.180,0.616"]
"Gestational age" [pos="0.427,0.580"]
"Hypertensive disorders" [pos="0.859,0.344"]
"Insurance status" [pos="0.234,0.099"]
"Maternal age" [pos="0.516,0.201"]
"Resource use in delivery period" [outcome,pos="0.644,0.910"]
ARFS [exposure,pos="0.264,0.908"]
Diabetes [pos="0.739,0.178"]
Parity [pos="0.680,0.385"]
Plurality [pos="0.783,0.735"]
Smoking [pos="0.098,0.457"]
"Alcohol drinking" -> "Assisted reproductive therapy"
"Alcohol drinking" -> Smoking
"Assisted reproductive therapy" -> "Gestational age"
"Assisted reproductive therapy" -> "Resource use in delivery period"
"Assisted reproductive therapy" -> Plurality
"BMI pre-pregnancy" -> "Hypertensive disorders"
"BMI pre-pregnancy" -> "Resource use in delivery period"
"BMI pre-pregnancy" -> Diabetes
"Education status" -> "Alcohol drinking"
"Education status" -> "Assisted reproductive therapy"
"Education status" -> "BMI pre-pregnancy"
"Education status" -> "Insurance status"
"Education status" -> "Maternal age"
"Education status" -> ARFS
"Education status" -> Parity
"Education status" -> Smoking
"Gestational age" -> "Insurance status"
"Gestational age" -> "Resource use in delivery period"
"Hypertensive disorders" -> "Resource use in delivery period"
"Insurance status" -> "Assisted reproductive therapy"
"Insurance status" -> "Resource use in delivery period"
"Maternal age" -> "Alcohol drinking"
"Maternal age" -> "Assisted reproductive therapy"
"Maternal age" -> "BMI pre-pregnancy"
"Maternal age" -> "Hypertensive disorders"
"Maternal age" -> "Resource use in delivery period"
"Maternal age" -> Diabetes
"Maternal age" -> Parity
```

"Maternal age" -> Plurality
"Maternal age" -> Smoking
ARFS -> "BMI pre-pregnancy"
ARFS -> "Resource use in delivery period"
Diabetes -> "Resource use in delivery period"
Parity -> "BMI pre-pregnancy"
Parity -> "Hypertensive disorders"
Parity -> Diabetes
Plurality -> "Gestational age"
Plurality -> "Resource use in delivery period"
Smoking -> "Gestational age"
}

Minimal sufficient adjustment sets for estimating the total effect of BMI pre-pregnancy on Resource use in delivery period:

- *Education status*

Aim iv) Estimate the direct unconfounded effect of maternal diet quality on resource use during the delivery admission

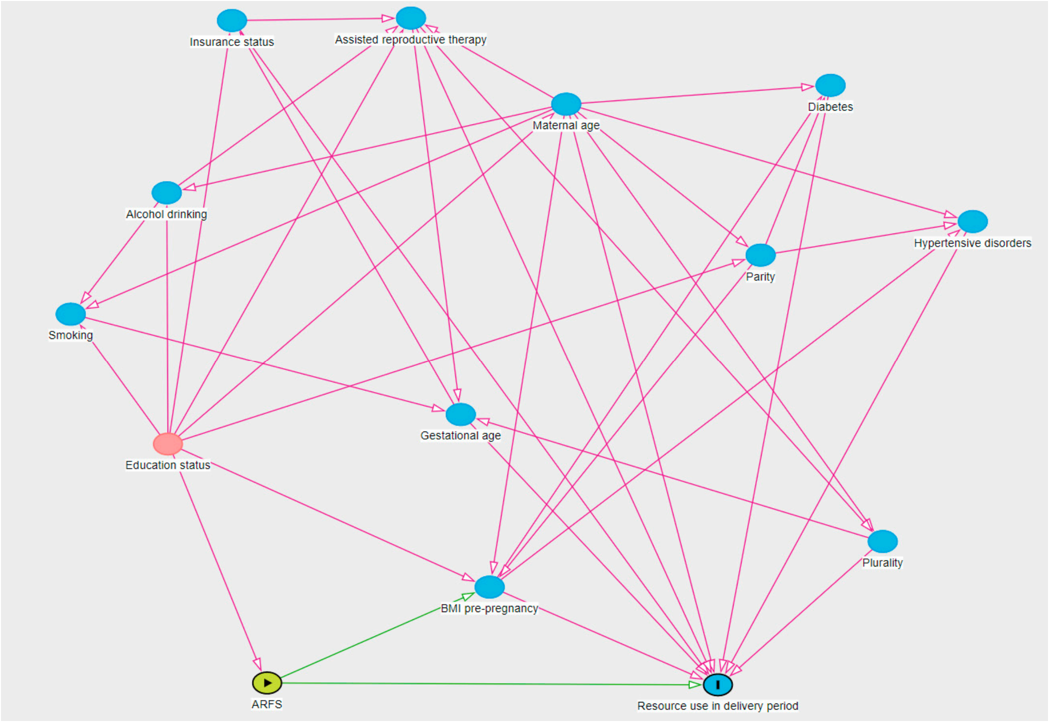

Figure 4. Directed acyclic graph developed for Aim (iv).
Each variable included in the DAG is listed in alphabetical order: 'Alcohol drinking indicates the participants alcohol consumption during this pregnancy; 'ARFS' indicates the participants diet quality; 'Assisted reproductive therapy' (ART) indicates the use for the current pregnancy; 'BMI pre-pregnancy' indicates the mothers BMI pre-pregnancy; 'Diabetes' indicates the participant's history of diabetes and diabetes status for the current pregnancy; 'Education status' indicates the mother's highest level of education attainment; 'Gestational age' indicates to the infants gestational age at birth; 'Hypertensive disorders' indicates the participant's history of hypertensive disorders and status for the current pregnancy; 'Insurance status' indicates a participant's insurance status for the birth admission (public or private patient); 'Maternal age' indicates the mothers age at delivery; 'Parity' indicates the number of pregnancies the participant has had; 'Resource use in the delivery period' indicates the participants resource use for the delivery admission; 'Smoking' indicates the participants smoking status during this pregnancy. Evidence to support the inclusion of each variable in this DAG is include in manuscript Appendix A. Table 5.

To generate the DAG in Figure 3. Launch DAGitty in your browser (http://www.dagitty.net/) and use the following model code:

```
dag {
bb="0,0,1,1"
"Alcohol drinking" [pos="0.179,0.309"]
"Assisted reproductive therapy" [pos="0.385,0.096"]
```

```
"BMI pre-pregnancy" [pos="0.451,0.791"]
"Education status" [pos="0.180,0.616"]
"Gestational age" [pos="0.427,0.580"]
"Hypertensive disorders" [pos="0.859,0.344"]
"Insurance status" [pos="0.234,0.099"]
"Maternal age" [pos="0.516,0.201"]
"Resource use in delivery period" [outcome,pos="0.644,0.910"]
ARFS [exposure,pos="0.264,0.908"]
Diabetes [pos="0.739,0.178"]
Parity [pos="0.680,0.385"]
Plurality [pos="0.783,0.735"]
Smoking [pos="0.098,0.457"]
"Alcohol drinking" -> "Assisted reproductive therapy"
"Alcohol drinking" -> Smoking
"Assisted reproductive therapy" -> "Gestational age"
"Assisted reproductive therapy" -> "Resource use in delivery period"
"Assisted reproductive therapy" -> Plurality
"BMI pre-pregnancy" -> "Hypertensive disorders"
"BMI pre-pregnancy" -> "Resource use in delivery period"
"BMI pre-pregnancy" -> Diabetes
"Education status" -> "Alcohol drinking"
"Education status" -> "Assisted reproductive therapy"
"Education status" -> "BMI pre-pregnancy"
"Education status" -> "Insurance status"
"Education status" -> "Maternal age"
"Education status" -> ARFS
"Education status" -> Parity
"Education status" -> Smoking
"Gestational age" -> "Insurance status"
"Gestational age" -> "Resource use in delivery period"
"Hypertensive disorders" -> "Resource use in delivery period"
"Insurance status" -> "Assisted reproductive therapy"
"Insurance status" -> "Resource use in delivery period"
"Maternal age" -> "Alcohol drinking"
"Maternal age" -> "Assisted reproductive therapy"
"Maternal age" -> "BMI pre-pregnancy"
"Maternal age" -> "Hypertensive disorders"
"Maternal age" -> "Resource use in delivery period"
"Maternal age" -> Diabetes
"Maternal age" -> Parity
```

```
"Maternal age" -> Plurality
"Maternal age" -> Smoking
ARFS -> "BMI pre-pregnancy"
ARFS -> "Resource use in delivery period"
Diabetes -> "Resource use in delivery period"
Parity -> "BMI pre-pregnancy"
Parity -> "Hypertensive disorders"
Parity -> Diabetes
Plurality -> "Gestational age"
Plurality -> "Resource use in delivery period"
Smoking -> "Gestational age"
}
```

Minimal sufficient adjustment sets for estimating the direct effect of ARFS on Resource use in delivery period:

- *Assisted reproductive therapy, BMI pre-pregnancy, Diabetes, Gestational age, Hypertensive disorders, Insurance status, Maternal age, Plurality*
- *Assisted reproductive therapy, BMI pre-pregnancy, Diabetes, Gestational age, Hypertensive disorders, Insurance status, Maternal age, Smoking*
- *Assisted reproductive therapy, BMI pre-pregnancy, Gestational age, Insurance status, Maternal age, Parity, Plurality*
- *Assisted reproductive therapy, BMI pre-pregnancy, Gestational age, Insurance status, Maternal age, Parity, Smoking*
- *BMI pre-pregnancy, Diabetes, Education status, Hypertensive disorders, Maternal age*
- *BMI pre-pregnancy, Education status, Maternal age, Parity*
